# Editorial: Social norms in managerial decision-making: Psychological and/or neural perspectives

**DOI:** 10.3389/fpsyg.2022.1099149

**Published:** 2023-01-05

**Authors:** Xile Yin, Jianbiao Li, Dahui Li, Yufei Ren, Guangrong Wang

**Affiliations:** ^1^School of Business Administration, Zhejiang Gongshang University, Hangzhou, China; ^2^School of Economics, Shandong University, Jinan, China; ^3^Labovitz School of Business and Economics, University of Minnesota Duluth, Duluth, MN, United States; ^4^School of Economics and Management, Weifang University, Weifang, China

**Keywords:** social norms, innovation, donation, social preference, pro-environmental behavior, corporate social responsibility (CSR)

## Introduction

Social norms, the concept that originates from the field of sociology (Opp, 1982; Elster, 1989), represent what people ought to do or actually do in a specific situation (Cialdini et al., 1990; Bonan et al., 2020; Yin et al., 2021). As Fehr and Fischbacher (2004, p. 63) stated that “the ability to develop and enforce social norms is probably one of the distinguishing characteristics of the human species,” social norms can greatly influence individual and organizational decision-making processes.

The impact of social norms has long been a core topic in the fields of behavioral economics, psychology, sociology, and decision neuroscience. There is also increasing attention from studies of managerial decision-making in recent years. For instance, several reviews and Editorial articles have emphasized the usefulness of social norm theory in empirical business ethics research (Blay et al., 2018) and corporate governance (Stathopoulos and Talaulicar, 2022). However, the discussion on management issues is insufficient. We still know little about how social norms affect specific managerial decision-makings, the management systems and forces that constrain or enhance social norms, the cognitive and emotional mechanisms in the process, as well as the relevant neural evidence. To address this important while underexplored research area, we have proposed this Research Topic and finally accepted 13 manuscripts. These manuscripts have investigated the roles of social norms in managerial decisions such as donation and pro-social activities (five articles), corporate social responsibility (CSR) and pro-environmental behaviors (four articles), and corporate innovation (four articles). The main ideas and contributions of these articles are outlined below.

## Social norms in donation decisions and pro-social activities

Several papers have examined the roles of social norms in donation decisions. Prior studies have shown that donors who are informed of information about other people' previous donation tend to comply with social norms by mimicking these people's donation decisions (Smith et al., 2015; Drouvelis and Marx, 2021). Peng et al. investigated how individual donation behavior was affected by previous information and found that donors imitated not only the amount of donated money but also the choice of anonymity and the positive sentiment expressed by others.

Similarly, the article from Li et al. explored the influences of social capital and social recommendation on charity crowdfunding performance. Using 4,780 project data from the charity crowdfunding of Sina MicroBlog, the authors found that both external social capital and internal capital significantly improved the fundraising performance of crowdfunding projects. Moreover, projects with more social recommendations were more likely to obtain financial support.

As a study focusing on the impact factors of corporate philanthropic donations, Chen H. et al. used data from the 12th Chinese Privately Owned Enterprise (POEs) Survey and found that an entrepreneur's military experience had a positive influence on corporate philanthropic donations. Further, the entrepreneur with military experience still donated even if their firms suffered from financial constraints. The findings by Chen H. et al. suggest that entrepreneurs with military experience may be more likely to value social norms for altruism.

Additionally, two articles investigated the roles of social norms in pro-social activities. Using an experimental design of the sequential public goods game, Fu et al. showed that the level of a leader's investment in public goods could significantly affect the cooperation behavior of team members. The results of Wang et al. demonstrated an inverted *U*-shaped relationship between top management team (TMT) compensation gap and corporate performance, and this relationship was weakened when TMT members owned a higher level of fairness preference.

## Social norms in CSR and pro-environmental behaviors

There are several papers investigating the roles of social norms in CSR. Wang and Cao investigated executives' decision-making on CSR activities between their predecessor firms and the successor firms by tracking their movements across Chinese listed firms, which indicated that their value for social norms for CSR maintained consistently in a certain period. Moreover, two other articles by Khan et al. and Qu et al. examined the influence of tournament incentives on CEOs and corporate network position on CSR, respectively, which showed that the above two factors demonstrated positive effects on CSR Performance.

Additionally, Wan and Deng presented an experimental study of the effect of group identity on pro-environmental behaviors. They found that group identity primed by housing ownership did not affect individual environmental behavior, while social norms primed by publicity and education showed significant positive effects on the development of individual and group pro-environmental behavior.

## Social norms in corporate innovation

Four articles introduced the influences of social norms on corporate innovation activities. Two articles suggested that enterprise managers might treat typical innovation activities of market stakeholders (e.g., upstream enterprises, downstream enterprises, and competitors) as social norms, and then conducted similar innovation activities for their own enterprises. For instance, Chen S. et al. presented an empirical study of the influences of external innovations from upstream enterprises, downstream enterprises, and competitors on the exports of private enterprises. They found that the external innovations by market shareholders could significantly promote private enterprises' innovations, which further enhanced their export performance. Similarly, Liu et al. also found the existence of peer effect in the innovation activities of listed enterprises.

Additionally, Zhang and Ma investigated the relationship between faultlines of board directors and innovation activities of Chinese companies. The results showed that social-related faultlines demonstrated a negative effect on corporate innovation while cognitive-related faultlines showed a positive effect. The authors suggested that social-related faultlines might lead to the out-group discrimination effect among sub-groups of the board, and thus harmed corporate innovation performance.

Finally, Shao et al. introduced an empirical study which investigated the roles of celebrity CEOs in firms' innovation investment activities. The authors suggested that the norms for preserving celebrity status would motivate celebrity CEOs to develop a higher level of innovation investments. The results also showed that the effect of celebrity CEOs on innovation investment was positively moderated by analyst coverage. These findings were consistent with the ideas that social norms would show a higher influence when subjects were under observation by social members (Schram and Charness, 2015).

## Conclusions

This Research Topic highlights the roles of social norms in managerial decision-making. The collected articles demonstrate that social norms can influence a wide scope of business activities, ranging from donation, CSR, pro-environmental behaviors to corporate innovation. We suggest that future lines of research can further explore how social norms interact with country- and firm-level institutional characteristics to influence business activities in different countries.

## Author contributions

All authors listed have made a substantial, direct, and intellectual contribution to the work and approved it for publication.
